# Total HIV/AIDS Expenditures in Dehong Prefecture, Yunnan Province in 2010: The First Systematic Evaluation of Both Health and Non-Health Related HIV/AIDS Expenditures in China

**DOI:** 10.1371/journal.pone.0068006

**Published:** 2013-06-25

**Authors:** Duo Shan, Jiangping Sun, Anna Yakusik, Zhongdan Chen, Jianhua Yuan, Tao Li, Jeannia Fu, Kaveh Khoshnood, Xing Yang, Mei Wei, Song Duan, Marc Bulterys, Michael Sante, Runhua Ye, Lifen Xiang, Yuecheng Yang

**Affiliations:** 1 National Center for AIDS/STD Control and Prevention, Chinese Center for Disease Control and Prevention, Beijing, China; 2 UNAIDS Belarus office, Minsk, Belarus; 3 UNAIDS China office, Beijing, China; 4 Beijing Information and Control Institute, Beijing, China; 5 School of Public Health, Yale University, New Haven, Connecticut, United States of America; 6 Dehong Prefecture Health Bureau, Dehong, Yunnan, China; 7 Dehong Prefecture Center for Disease Control and Prevention, Dehong, Yunnan, China; 8 Global AIDS Program China office, Beijing, China; Rollins School of Public Health, Emory University, United States of America

## Abstract

**Objective:**

We assessed HIV/AIDS expenditures in Dehong Prefecture, Yunnan Province, one of the highest prevalence regions in China, and describe funding sources and spending for different categories of HIV-related interventions and at-risk populations.

**Methods:**

2010 HIV/AIDS expenditures in Dehong Prefecture were evaluated based on UNAIDS’ National AIDS Spending Assessment methodology.

**Results:**

Nearly 93% of total expenditures for HIV/AIDS was contributed by public sources. Of total expenditures, 52.7% was allocated to treatment and care, 24.5% to program management and administration and 19.8% to prevention. Spending on treatment and care was primarily allocated to the treatment of opportunistic infections. Most (40.4%) prevention spending was concentrated on most-at-risk populations, injection drug users (IDUs), sex workers, and men who have sex with men (MSM), with 5.5% allocated to voluntary counseling and testing. Prevention funding allocated for MSM, partners of people living with HIV and prisoners and other confined populations was low compared to the disproportionate burden of HIV/AIDS in these populations. Overall, people living with HIV accounted for 57.57% of total expenditures, while most-at-risk populations accounted for only 7.99%.

**Conclusions:**

Our study demonstrated the applicability of NASA for tracking and assessing HIV expenditure in the context of China, it proved to be a useful tool in understanding national HIV/AIDS response from financial aspect, and to assess the extent to which HIV expenditure matches epidemic patterns. Limited funding for primary prevention and prevention for MSM, prisoners and partners of people living with HIV, signal that resource allocation to these key areas must be strengthened. Comprehensive analyses of regional and national funding strategies are needed to inform more equitable, effective and cost-effective HIV/AIDS resource allocation.

## Introduction

A joint surveillance effort undertaken by the China Ministry of Health, UNAIDS, and WHO in 2011 estimated that there are currently 780,000 (0.058%) individuals living with HIV/AIDS in China, with the majority having been infected through heterosexual contact (46.5%), followed by injection drug use (IDU) (28.4%), male homosexual contact (17.4%), contaminated blood product (6.6%), and mother-to-child transmission (1.1%). HIV incidence has also been steadily increasing, with 48,000 new infections in 2009. Nearly 76% of all individuals living with HIV/AIDS remain concentrated in six primary provinces, including Yunnan, Guangxi, Henan, Sichuan, Xinjiang, and Guangdong [1].

Given the rapidly growing national HIV/AIDS epidemic, which demands significant expansion of HIV treatment and prevention efforts, the government has confronted new concerns related to funding and resource allocation for HIV/AIDS. Efforts to assess whether current funding for HIV/AIDS is being allocated effectively and cost-effectively and is sufficient to meet growing treatment and prevention needs are urgently needed, particularly among specific sub-populations at risk and in the regions most affected by the HIV/AIDS epidemic. In order to undertake these assessments, comprehensive, systematic description of HIV/AIDS expenditures, by funding source, intervention category, and populations targeted, is required. However, systematic evaluations of HIV/AIDS expenditures in China that include both health and non-health related expenditures have yet to be reported in the peer-reviewed literature. These data are needed to inform both future funding strategies and cost-effectiveness analyses that can guide decision-making in HIV/AIDS resource allocation.

Since the first HIV cases were detected among people who inject drugs in Dehong Prefecture in Yunnan Province in 1989, HIV/AIDS prevention and control work in this region has received extensive domestic and international attention. The HIV epidemic in Dehong continues to be one of the largest in China, with 17,590 cumulative reported HIV/AIDS cases at the end of 2010, representing 6.4% of China’s reported HIV/AIDS cases despite constituting only 0.08% of China’s total population [2]. IDUs continue to represent the largest group of HIV-infected individuals in Dehong Prefecture. In recent years, however, sexual transmission has overtaken IDU as the most commonly reported mode of HIV transmission in Yunnan Province. Surveillance data demonstrate that HIV prevalence among injecting drug users (IDUs) was 31.1% in 2010, 28.6% among men having sex with men, 13.8% among HIV-discordant couples, 1.7% among female sex workers, 0.7% among pregnant women (0.1%), and 0.8% among pre-marital couples. Paralleling these patterns, the estimated annual HIV incidence among injecting drug users (IDUs) was 4.3%, 4.7% in HIV discordant couples, 1.3% in female sex workers, 0.1% in pregnant women, and 0.1% in pre-marital couples [3,4]. In 2004, Dehong Prefecture was designated by the Ministry of Health as one of 4 key HIV/AIDS prevention and control key sites. Given the disproportionate burden of HIV infection in the region, Dehong has consistently received comparatively large funding allocations from the central government. A total of RMB 20,700,000 ($3,057,787.76) in 2008 and RMB 40,390,000 ($5,966,379.11) in 2010 were allocated specifically to Dehong. Funding overall has come from a wide array of sources, including the central, provincial and prefectural government, international agencies, and from national scientific research funding.

Given the state of the HIV/AIDS epidemic in Dehong Prefecture and the concentration of funding for HIV prevention and control efforts in this region, current HIV/AIDS expenditures require careful description so that policymakers and other actors can be better evaluate the equity, efficiency and effectiveness of existing resource allocation patterns [5]. In this study, we undertake a systematic evaluation of total HIV/AIDS expenditures in both health and non-related sectors in Dehong Prefecture in 2010, the first evaluation of this kind conducted in China.

## Materials and Methods

### National AIDS Spending Assessment (NASA) Tool

National AIDS Spending Assessment (NASA) tool was developed by UNAIDS in 2006 to track actual HIV/AIDS expenditures (public, private and international) in both health and non-health related sectors (e.g. social mitigation, education, labor, and justice) involved in national HIV/AIDS responses. NASA evaluates expenditure flow on three primary dimensions, finance, provision and consumption, thereby tracing the flow of funds from start to finish, and identifies expenditures in six general categories:

◆ Financing sources

◆ Financing agents

◆ Providers of HIV services

◆ Production factors

◆ AIDS spending categories

◆ Beneficiary populations

Eight AIDS spending categories include:

◆ Prevention

◆ Treatment and care

◆ Orphans and vulnerable children

◆ Programme management and administration

◆ Human resources

◆ Social protection and social services

◆ Enabling environment and community programs

◆ HIV-related research

NASA differs from other national HIV/AIDS expenditure categorization tools in that it includes assessment of both health- and non-health related expenditures [6]. Presently, NASA methodology and classifications have been applied in the assessment of HIV/AIDS expenditures in several countries and regions worldwide [7–12].

### Data Collection

**Figure 1:**
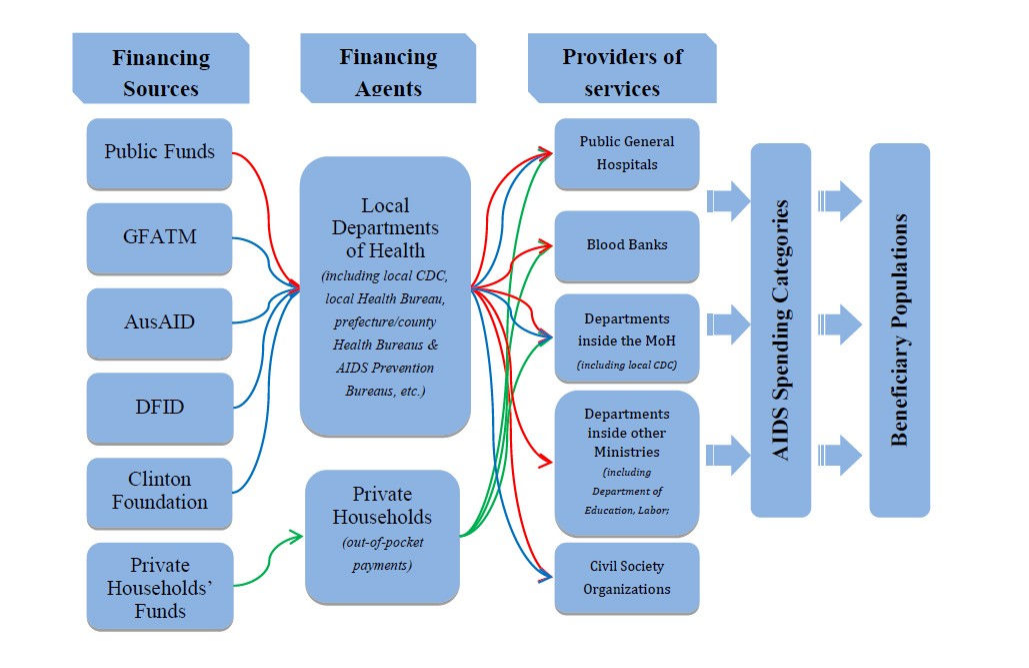
Flow of HIV/AIDS expenditures in Dehong Prefecture, 2010.

For this study, NASA categorizations were adapted and defined with regard to the local context and local organizational structures through consultation with UNAIDS and interviews with local programmatic and financial managers from healthcare institutions utilizing HIV/AIDS funds. We used the NASA tool to evaluate the allocation and flow of HIV/AIDS-related expenditures in Dehong Prefecture in 2010 (See Figure 1).

A NASA survey questionnaire was adapted to the local context and administered to providers. Providers included programmatic and financial managers from all the health care institutions utilizing HIV/AIDS funds, including 16 public general hospitals, 2 blood banks, 14 departments inside the local Ministry of Health (including the local health bureau and the local Centers for Disease Control), 38 departments within other ministries (including the Ministry of Education; Labor; Justice, etc.), and 28 civil society organizations. Survey content included local background data (HIV infection and population data), information from providers’ financial records (e.g. funds received, transferred, and used by the providers in 2010), provider expenditures for spcific HIV/AIDS prevention and control activities (including detailed data on funding sources, project descriptions, funds allocated to specific projects, populations targeted and number of participants reached). Altogether, 196 providers were surveyed.

### Statistical Analysis

Data was analyzed suing RTS v 2009.3.0 (Resource Tracking System Version 2009.3.0e), a data input software specifically developed by UNAIDS to input data collected for NASA. SPSS13.0 software was used for summary and analysis of NASA data exported using the RTS software.

### Ethics Statement

The study received approval from the institutional review board (IRB) of the National Center for AIDS/STD Control & Prevention, Chinese CDC.

As our study focused on the financial allocation and flows in 2010, and local financial records were provided by the study subjects (programmatic and financial managers from health care institutions), this study did not involve information of people living with HIV/AIDS, or patients. As to the population-based prevalence data used in this study, there was no name in the course of data collection and analysis. Thus, there was no informed consent in our study as it did not involve patients themselves or their information stored in the hospital database.

## Results

### HIV/AIDS Funding Sources

Total HIV/AIDS expenditures in Dehong prefecture in 2010 were estimated to be $ 6,773,498.25 and per capita spending was estimated to be $5.64 ($ 6,773,498.25/1,200,000). Public sources were estimated to have accounted for 92.7% ($ 6,278,129.74) of all funding with international sources accounting for 6.8% ($460,422.72) and private sources ($34,945.79) accounting for 0.5% (See Table 1).

**Table 1:** HIV/AIDS funding sources in Dehong Prefecture in 2010.

Sources of Funding	Amount (CNY/RMB)	Amount (US$)*	Proportions (%)
**Public funds**	42,500,427.12	6,278,129.74	92.69
Central government revenue	38,546,632.12	5,694,078.25	84.06
Local/municipal government revenue	3,733,290.00	551,478.67	8.14
State/provincial government revenue	220,505.00	32,572.83	0.48
**Private funds**	236,569.00	34,945.79	0.52
Households’ funds	149,809.00	22,129.67	0.33
Profit-making institutions and corporations	86,760.00	12,816.12	0.19
**International funds**	3,116,877.65	460,422.72	6.8
Government of Australia	326,447.00	48,222.49	0.71
Government of the United Kingdom	59,977.00	8,859.76	0.13
The Clinton Foundation	125,429.00	18,528.27	0.27
The Global Fund to Fight AIDS, Tuberculosis and Malaria	2,605,024.65	384,812.20	5.68
**Total expenditure**	45,853,873.77	6,773,498.25	100

* US$1 = ¥6.7696 Chinese Renminbi Yuan

### HIV/AIDS Spending Categories (SeeTable 2)

An estimated 52.6% of total expenditures was allocated to HIV treatment and care, followed by 24.5% to program management and administration, 19.8% to HIV prevention, human resources (2.75%), Social protection and social services (0.29%), Orphans and vulnerable children (OVC) (0.09%), Enabling environment (0.05%) and HIV-related research (0.01%).

**Table 2:** 2010 HIV/AIDS expenditures in Dehong Prefecture, by spending category.

Key intervention areas	Public funds (US $)	Private funds (US $)	International funds (US $)	Total expenditure (US $)	Proportions (%)
Prevention	1,124,566.52	2,658.95	212,345.74	1,339,571.20	19.78
Care and treatment	3,442,349.52	19,470.72	100,017.57	3,561,837.81	52.58
Orphans and vulnerable children (OVC)	1,624.91	0	4,234.37	5,859.28	0.09
Programme management and administration	1,514,616.27	12,816.12	128,799.93	1,656,232.32	24.45
Human resources	171,036.25	0	15,025.11	186,061.36	2.75
Social protection and social services*	3,692.98	0	0	3,692.98	0.05
Enabling environment	590.88	0	0	590.88	0.01
HIV-related research**	19,652.42	0	0	19,652.42	0.29
Total expenditure	6,278,129.75	34,945.79	460,422.72	6,773,498.25	100

* excluding OVC

** excluding operational research

#### Expenditures in HIV Treatment and Care

Public funding constituted 96.6% of all total expenditures in HIV treatment and care. Inpatient treatment of opportunistic infections accounted for the majority (42.1%; $ 1499853.31) of all spending on treatment and care, followed by antiretroviral therapy (30.4%; $ 1084104.53), outpatient prophylaxis and treatment of opportunistic infections (8.17%; $290940.38), provider-initiated testing and counseling (PITC) (2.15%; $76424.35), specific HIV-related laboratory monitoring (12.60%; $448852.37) and psychological treatment and support services (2.45%; $87178.41).

#### Expenditures in HIV Prevention

As with treatment and care, public funding constituted the majority of all total expenditures in HIV prevention (83.9%). However, compared to treatment and care spending, there was higher proportional contribution from international sources (15.9%). Prevention spending was focused primarily on ‘most at-risk populations’ (40.4%), including injection drug users (IDUs), sex workers, and men who have sex with men (MSM), followed by prevention of mother-to-child transmission (26.7%), risk-reduction for vulnerable and accessible populations (9.3%), including indigenous groups, truck drivers, prisoners, and migrants), public and commercial sector male condom provision (6.5%; $86,567.60), voluntary counseling and testing (VCT) (5.5%; $73,278.16), communication for social and behavior change (5.4%; 72,659.39), prevention of HIV transmission aimed at people living with HIV (1.9%; $25,868.00), blood safety (1.2%; $15,839.99), and the prevention, diagnosis, and treatment of sexually transmitted infections (STIs) (1.1%; $14,771.92). Of the most at-risk populations, expenditures were highest for IDUs (17.6%) and lowest for MSM (7.0%) (See Table 3).

**Table 3:** 2010 HIV/AIDS expenditures in HIV Prevention in Dehong Prefecture, by spending category.

**Prevention by spending category**	**Public funds (US$)**	**Private funds (US$)**	**International funds (US$)**	**Total (US$)**	**Proportions (%)**
Communication for social and behaviour change	71,204.35	0.00	1,455.03	72,659.39	5.42
Community mobilization	792.07	0.00	4,372.49	5,164.56	0.39
Voluntary counselling and testing (VCT)	73,278.16	0.00	0.00	73,278.16	5.47
Risk-reduction for vulnerable and accessible populations	79,707.69	0.00	44,597.80	124,305.50	9.28
Prevention – youth in school	9,111.32	0.00	0.00	9,111.32	0.68
Prevention – youth out-of-school	7,533.68	0.00	0.00	7,533.68	0.56
Prevention of HIV transmission aimed at people living with HIV (PLHIV)	22,262.91	0.00	3,605.09	25,868.00	1.93
Prevention programmes for MARP (IDU, SW, MSM)	397,459.64	0.00	143,859.08	541,318.72	40.41
Prevention programmes for IDU	167,570.62	0.00	68,444.34	236,014.96	17.62
Prevention programmes for FSW	146,415.89	0.00	65,781.05	212,196.94	15.84
Prevention programmes for MSM	83,473.13	0.00	9,633.69	93,106.82	6.95
Prevention programmes in the workplace	4,431.58	0.00	0.00	4,431.58	0.33
Condom social marketing	443.16	0.00	0.00	443.16	0.03
Public and commercial sector male condom provision	86,567.60	0.00	0.00	86,567.60	6.46
Prevention, diagnosis, and treatment of sexually transmitted infections (STI)	14,771.92	0.00	0.00	14,771.92	1.10
Prevention of mother-to-child transmission (PMTCT)	340,348.51	2,658.95	14,456.25	357,463.71	26.68
Blood safety	15,839.99	0.00	0.00	15,839.99	1.18
Post-exposure prophylaxis (PEP)	369.30	0.00	0.00	369.30	0.03
Prevention activities not broken down by intervention	444.63	0.00	0.00	444.63	0.03
Total expenditure	1,124,566.52	2,658.95	212,345.74	1,339,571.20	100.00

### Expenditures among specific beneficiary populations

**Figure 2:**
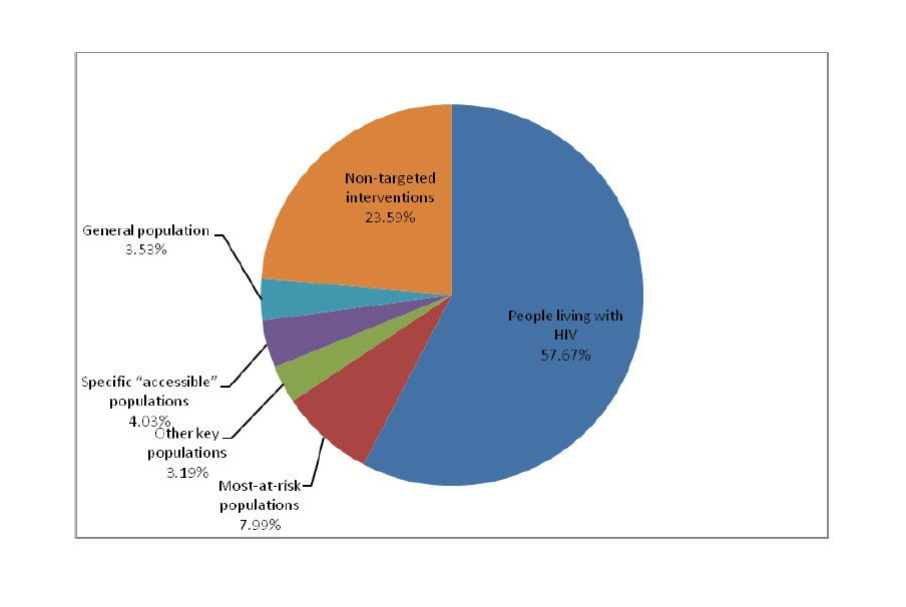
2010 HIV/AIDS Beneficiary Populations in Dehong Prefecture.

People living with HIV accounted for 57.7% ($3,906,082.92) of total expenditures, non-targeted populations (populations not targeted directly by interventions or only indirectly benefiting from interventions) accounted for 23.6%, while most at-risk populations (IDUs, FSW, MSM) accounted for only 8.0% (See Figure 2).

### Comparison between HIV prevalence and proportional spending for HIV/AIDS beneficiary populations

**Figure 3:**
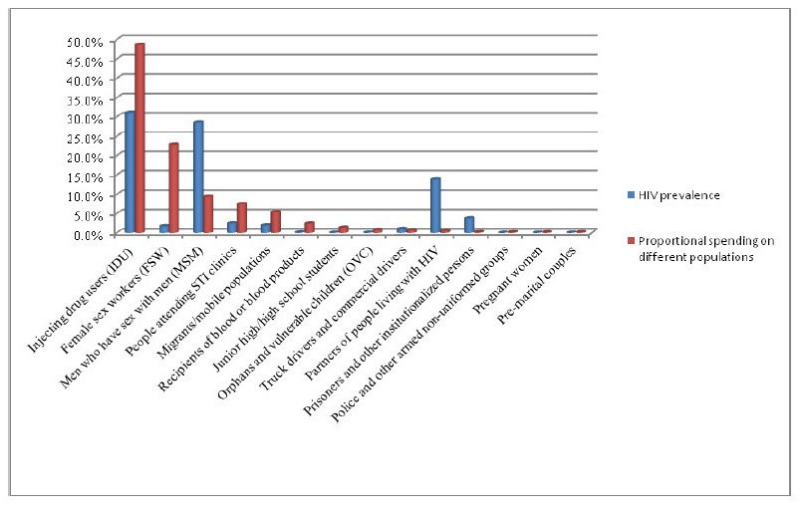
Comparison between HIV prevalence and proportional spending for HIV/AIDS beneficiary populations in Dehong Prefecture in 2010.

We compared proportional spending for specific populations to the reported HIV prevalence in these populations in 2010 (See Figure 3) and found that funding did not appear to be allocated proportionally to the burden of HIV/AIDS cases across all of the highest prevalence groups.

## Discussion

This is the first study to use systematic methods to evaluate both health and non-health related HIV/AIDS expenditures in China. We found that in 2010, while MSM constituted 28.6% of all HIV/AIDS cases in 2010, they represented only 9.4% of all HIV/AIDS spending. Similarly, partners of people living with HIV constituted 13.8% of all HIV/AIDS cases in 2010 yet represented 0.5% of total spending, and prisoners and other confined populations constituted 3.8% of all HIV/AIDS cases in 2010 yet only represented 0.2% of all spending. Most funds were used in treatment and care, followed by programme management and administration, and HIV prevention. The concentration of treatment funding on treatment of opportunistic infections, rather than on antiretroviral therapy, suggest that efforts to diagnose and link individuals to treatment and care earlier in the course of their infection may significantly reduce these costs.

In 2010, the HIV/AIDS response in Dehong Prefecture was predominantly funded by the central government, which has made a strong financial commitment to addressing the growing regional HIV epidemic. International funding sources had a limited contribution to overall expenditures, which may be expected given that international funding is largely funneled to higher-levels of operation [13,14].

With regard to overall HIV/AIDS expenditures, while over half of all funds were allocated to treatment and care, nearly a quarter of all funds were spent on management and administration. In a study by Aran-Matero et al. evaluating levels of spending and resource allocation to HIV programs in Latin Americ and the Caribbean, among the 20 countries evaluated, program management and administration strengthening accounted for only 4% of overall HIV/AIDS expenditures, in comparison to our finding of 24% in this study [15]. Program management and administration clearly constituted a disproportionately large proportion of total spending, underscoring the need to improve efficiency and coordination in this area so that increased funding can be allocated to the actual provision of treatment and prevention services. Improved service integration may be an important approach to reducing potentially redundant managerial and administrative costs.

We found limited funding for voluntary HIV testing and counseling and, surprisingly, found greater allocation to the treatment of inpatient and outpatient treatment of opportunistic infections than to antiretroviral therapy. Collectively, inpatient and outpatient treatment of opportunistic infections totaled to 50.3% of total expenditures on treatment and care. A 2011 study by Fujie Zhang et al suggested that initiating highly active antiretroviral therapy in patients with higher CD4 counts could potentially reduce treatment expenses related to opportunistic infections in immunocompromised individuals [16]. These findings along with the findings from this study suggest that by increasing efforts to diagnose and link individuals to treatment and care earlier in the course of their infection, and thereby funding of these initiatives, costs related to the treatment of opportunistic infections, which currently account for the majority of treatment and care spending, may become greatly reduced.

Importantly, the paucity of funds allocated to prevention in at-risk populations such as IDUs and MSM do not reflect the disproportionate burden of HIV infection in these populations. Demonstrating the smaller overall allocation to HIV prevention, people living with HIV accounted for 57.57% of total HIV/AIDS expenditures, while the most-at-risk populations accounted for only 7.99%, despite accounting for the majority of overall prevention spending. Additionally, our findings demonstrate that funding has not been proportional to the burden of HIV infection in several important at-risk groups, with notable underspending among MSM, partners of people living with HIV, and prisoners and other confined populations. Resource allocation to both HIV treatment and prevention in these at risk populations must be strengthened and may ultimately help to reduce overall costs [17,18].

We have systematically described both health and non-health related HIV/AIDS expenditures in China at the prefecture-level and identified important resource allocation concerns that have clear implications for China’s ongoing HIV control efforts. Given the scale of China’s HIV/AIDS epidemic and rising HIV transmission among specific at-risk populations, these concerns urgently need to be addressed. Our study has demonstrated the feasibility of adapting the UNAIDS’ NASA methodology and classification to the Chinese context and its potential be applied more widely throughout China. In order to make meaningful and sustained reductions in the HIV/AIDS epidemic, systematic, comprehensive evaluation of China’s cumulative HIV/AIDS expenditures ultimately need to be conducted to inform more equitable, effective and cost-effective HIV/AIDS resource allocation at the national level.
